# Supplementary material for: Identification of photoperiod-induced specific miRNAs in the adrenal glands of Sunite sheep (Ovis aries)
Source: Front Vet Sci. 2022 Jul 22;9:888207. doi: 10.3389/fvets.2022.888207 (PMC9354845; doi:10.3389/fvets.2022.888207)
Supplement: Supplementary file 1 [file Data_Sheet_1.ZIP › Supplementary file/miRDeep2 file.zip!/miRDeep2 file/Know_Structure/SPLP42A1/oar-mir-544.pdf]

miRBase precursor : oar-mir-544
Total read count : 496
oar-miR-544-5p read count : 466
oar-miR-544-3p read count : 22
remaining reads : 8

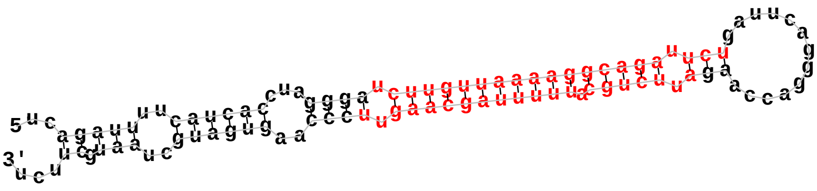

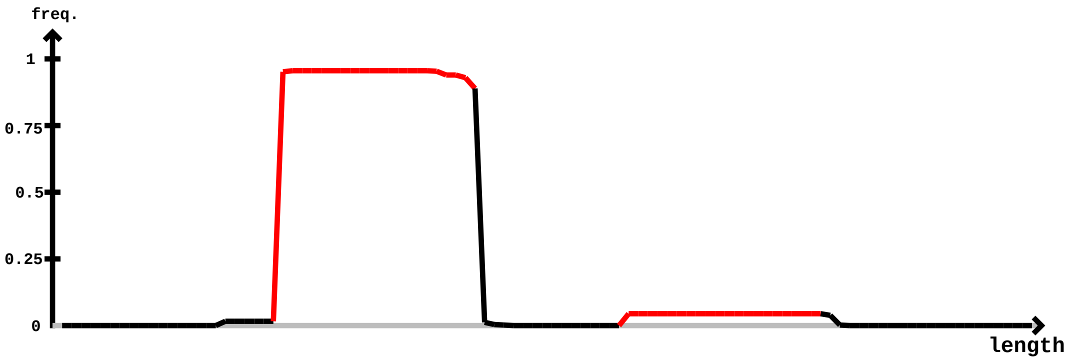

oar-miR-544-5p

oar-miR-544-3p

```
5'- ucagauuuucaucaccuagggaucuuguuaaaaggcagauucugauucagggaccaagauucugcauuuuuagcaaguucccaagugaugcuaaugcuucu   -3'    exp
    ..(((((..((((((...((((.((((((((((((((((.(((.............))).))))).))))))))))).))))..))))))..))).))...    reads   mm   sample
    ................uagggaucuuguuaaaaggcag...............................................................    1       0    seq
    ................uagggaucuuguuaaaaggcaga..............................................................    7       0    seq
    ......................ucuuguuaaaaggcagauu............................................................    4       0    seq
    ......................ucuuguuaaaaggcagauuU...........................................................    1       1    seq
    ......................ucuuguuaaaaggcagauuc...........................................................    19      0    seq
    ......................ucuuguuaaaaggcagaCucu..........................................................    1       1    seq
    ......................ucuuguuaaaaggcagauucu..........................................................    399     0    seq
    ......................Ccuuguuaaaaggcagauucu..........................................................    3       1    seq
    ......................ucuuguuaaaaggcagGuucu..........................................................    2       1    seq
    ......................ucuuguuaaaaggcagauuUu..........................................................    1       1    seq
    ......................ucuuguuaaaaggcagUuucu..........................................................    1       1    seq
    ......................uUuuguuaaaaggcagauucu..........................................................    1       1    seq
    ......................ucuuAuuaaaaggcagauucu..........................................................    2       1    seq
    ......................ucuuguuaaaaggcagauucA..........................................................    23      1    seq
    ......................ucuuguCaaaaggcagauucu..........................................................    2       1    seq
    ......................ucuuguuaaaaggcagauucug.........................................................    1       0    seq
    ......................ucuuguuaaaaggcagauucuA.........................................................    1       1    seq
    ......................ucuuguuaaaaggcagauucuU.........................................................    2       1    seq
    ......................ucuuguuaaaaggcagauucugU........................................................    1       1    seq
    .......................cuuguuaaaaggcagauu............................................................    1       0    seq
    .......................cuuguuaaaaggcagauucugau.......................................................    1       0    seq
    ..........................................................auucugcauuuuuagcaagCu......................    1       1    seq
    ..........................................................auucugcauuuuuagcaaguu......................    2       0    seq
    ..........................................................auucugcauuuuuagcaaguuU.....................    5       1    seq
    ..........................................................auucugcauuuuuagcaaguuc.....................    13      0    seq
    ..........................................................auucugcauuuuuagcaaguucc....................    1       0    seq
```
